# Klebsiella ozaenae in a Peritonsillar Abscess: A Case Report

**DOI:** 10.7759/cureus.58499

**Published:** 2024-04-17

**Authors:** Lamis Halawani, Kamal Hanbazazah, Mohammed Alshanbari

**Affiliations:** 1 Otolaryngology-Head and Neck Surgery, King Fahd Armed Forces Hospital, Jeddah, SAU; 2 Otolaryngology-Head and Neck Surgery, East Jeddah Hospital, Jeddah, SAU

**Keywords:** multidrug resistant (mdr), atrophic rhinitis, klebsiella, quinsy, klebsiella ozaenae, peritonsillar abscess

## Abstract

A peritonsillar abscess (PTA) is an infection that primarily affects the peritonsillar space. The incidence is estimated to affect 30 per 100,000 individuals annually, with a higher prevalence between the 15- and 30-year-old age groups. The pathogenesis of a PTA is a crucial step in effective management and prevention. Typically, a PTA has a polymicrobial etiology, aerobic, and anaerobic bacteria of oral flora. Multiple papers in the literature have studied the incidence of *Klebsiella* species in PTA cases. However, few studies have isolated *Klebsiella ozaenae* in a PTA. We present a case of a 29-year-old patient who was admitted as a case of a PTA. He underwent an incision and drainage of the right PTA in the operation room. A significant amount of purulent fluid was drained with a positive culture of *K. ozaenae*.

## Introduction

A peritonsillar abscess (PTA) is an infection that primarily affects the peritonsillar space, which is a potential space located between the tonsillar capsule and the superior constrictor muscle of the pharynx. It is also known as quinsy. The incidence is estimated to affect 30 per 100,000 individuals annually, with a higher prevalence between the 15- and 30-year-old age groups. The most common presentation includes severe sore throat, dysphagia, and truisms, which are often accompanied by a systematic manifestation such as fever and malaise [1].

The pathogenesis of a PTA is a crucial step in effective management and prevention. Typically, a PTA has a polymicrobial etiology, aerobic, and anaerobic bacteria of oral flora. Most common organisms include group A β-hemolytic *Streptococcus*, *Staphylococcus aureus*, and anaerobic *Fusobacterium* species [2].

Several papers in the literature have studied the incidence of *Klebsiella *species in PTA cases. However, few studies have isolated *Klebsiella ozaenae* in a PTA.

We present a case of a 29-year-old patient who was admitted with a case of PTA with a positive culture of *K. ozaenae*.

## Case presentation

A 29-year-old Saudi male, otherwise healthy, presented to the emergency department with a five-day history of progressive sore throat associated with dysphagia and odynophagia. He had a positive history of fever, with an inability to open his mouth. There is no history of recurrent tonsillitis. On examination, he had a muffled sound and trismus. Oropharyngeal examination showed a right tonsillar bulge, with uvular deviation to the left side, and tonsils were erythematous with exudates bilaterally. Neck examination was insignificant, with no airway compromise on endoscopic examination.

Aspiration using a wide bore needle of the right PTA gave a total of 6 mL of purulent fluid. Initially, the patient was started on intravenous clindamycin, co-amoxiclav, and dexamethasone. Improvement was observed in a patient’s condition. Upon daily assessment, the patient clinically deteriorated with the worsening of his symptoms, and the oropharyngeal examination did not change. The result of the wound culture was multidrug-resistant (MDR) *K. ozaenae*.

Images

**Figure 1:**
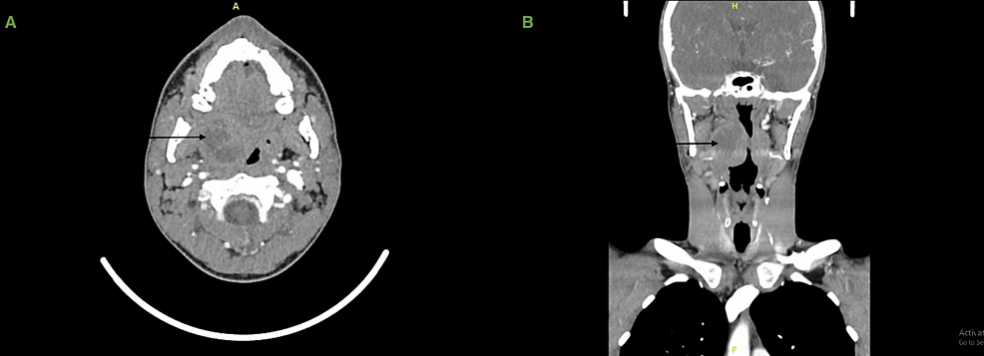
CT of the neck with IV contrast showing a well-defined collection in the right tonsillar region Axial view (A), coronal view (B)

An enhanced head and neck CT scan was performed during the admission after the patient's condition had deteriorated, and it showed a well-defined collection with peripheral enhancement noted in the right tonsillar region measuring 3.2x3.4x3.3 cm in posteroanterior (PA), transverse (TV), and craniocaudal (CC) diameters. Subsequent moderate to marked enhancement of the aerodigestive tract is noted with a contralateral midline shift, with no obstruction. Multiple regional right cervical lymph node enlargements were noted, with the largest located at the neck level IIA measuring 1 cm. No extension to adjacent structures (Figure 1).

Management

The patient underwent incision and drainage of the right PTA in the operation room under general anesthesia. A significant amount of purulent fluid was drained, and culture was taken, which resulted in no growth of organisms. The patient was started on tigecycline and imipenem based on culture and sensitivity and recommendations from an infectious diseases team, leading to significant clinical improvement. He was sent home in stable condition with further follow-up in the clinic.

## Discussion

Several studies have investigated the microbial etiology of a PTA, and while the *Streptococcus *species remain the most commonly isolated pathogens, emerging evidence suggests a notable contribution of *Klebsiella *species. The incidence of *Klebsiella *in PTA varies across geographical regions and patient populations [3].

A study conducted by Tsai et al. analyzed 415 cases of a PTA in Taiwan and found that *Streptococcus viridans* was the most common isolated bacteria (28.57%), followed by *Klebsiella pneumoniae* (23.21%) [3]. Another study by Slouka et al. focused on a different geographical area and reported a higher incidence of *Streptococcus pyogenes* and a lower incidence of *Klebsiella pneumoniae* [4]. These findings suggest that *Klebsiella *plays a significant role in the pathogenesis of a PTA than previously recognized. The prevalence may differ based on the patient’s demographics, local epidemiology, and underlying risk factors.

The identification of *Klebsiella *as a causative agent in a PTA has important clinical implications. Unlike the typical pathogens associated with a PTA, *Klebsiella *is a MDR pathogen, with a high morbidity and mortality rate. This resistance can lead to treatment failures and may require alternative therapeutic approaches, such as the use of carbapenems or combination therapies [5].

*K. ozaenae *is a species of bacteria commonly found in the environment, particularly in soil and water. It is a gram-negative bacillus and belongs to the family *Enterobacteriaceae*. *K. ozaenae *is primarily associated with a condition called ozaena, also known as atrophic rhinitis. This condition is characterized by crusts and foul-smelling discharge in the nasal passages [6,7]. However, its role in the pathogenesis of PTA remains poorly understood. *K. ozaenae *has been also found to be isolated in sputum [7], which could be the source of a PTA in our case.

The isolation of *K. ozaenae* could indicate impaired immune function, and the choice of antibiotics should be based on cultures and sensitivities [8].

## Conclusions

In conclusion, *K. ozaenae* is rarely isolated with, to our knowledge, and has not previously been reported to cause a PTA. Future studies should focus on elucidating the mechanisms by which *K. ozaenae* contributes to the development and progression of a PTA, facilitating the development of targeted therapeutic strategies.

Further research is warranted to elucidate the specific factors contributing to the incidence of *Klebsiella *in a PTA. A better understanding of the mechanisms of pathogenesis, host susceptibility, and epidemiology will enable targeted interventions to optimize patient care.
